# Cytotoxicity of Benzofuran-Containing Simplified Viniferin Analogues

**DOI:** 10.3390/ph17081012

**Published:** 2024-08-01

**Authors:** Salvatore Princiotto, Cecilia Pinna, Luce Micaela Mattio, Francesca Annunziata, Giovanni Luca Beretta, Andrea Pinto, Sabrina Dallavalle

**Affiliations:** 1 Department of Food, Environmental and Nutritional Sciences (DeFENS), University of Milan, Via Celoria 2, 20133 Milan, Italy; cecilia.pinna@unimi.it (C.P.); luce.mattio@unimi.it (L.M.M.); francesca.annunziata@unimi.it (F.A.); andrea.pinto@unimi.it (A.P.); sabrina.dallavalle@unimi.it (S.D.); 2 Molecular Pharmacology Unit, Department of Experimental Oncology, Fondazione IRCCS Istituto Nazionale Tumori, Via Amadeo 42, 20133 Milan, Italy; giovanni.beretta@istitutotumori.mi.it

**Keywords:** viniferin derivatives, dimeric stilbenoids, benzofuran nucleus, antiproliferative activity, DNA damage

## Abstract

Within the huge class of plant secondary metabolites, resveratrol-derived stilbenoids show wide structural diversity and mediate a great number of biological responses relevant for human health, including cancer prevention and cytotoxicity. Resveratrol is known to modulate several pathways directly linked to cancer progression, as well as its analogue pterostilbene, characterized by an increased metabolic stability and significant pharmacological activities. To study the potential anticancer activity of other stilbenoids, a home-made collection of resveratrol dimers and simplified analogues was tested on melanoma A375, non-small cell lung cancer H460 and PC3 prostate cancer cell lines. The structural determinants responsible for the antiproliferative activity have been highlighted. Moreover, to investigate the DNA damage ability of the selected molecules, the expression of the γ-H2AX after compound exposure was evaluated.

## 1. Introduction

**Figure 1:**
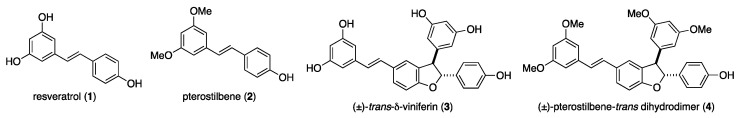
Structures of resveratrol, pterostilbene and dimeric derivatives **3** and **4**.

Stilbenoids are an abundant and widely distributed family of natural compounds found in various plant species [1]. They have been largely studied in recent decades due to their bioactivities that range from cardioprotection to neuroprotection, including anti-diabetic and anti-inflammatory properties [2,3]. Several recent studies highlighted their potentiality in cancer prevention and treatment [4]. The results cover a myriad of models, from cell cultures to animal studies, as well as clinical human trials. Resveratrol (Figure 1) is the most deeply investigated stilbenoid, with considerable evidence supporting its anticancer properties [5]. In fact, it is known to modulate several pathways that are directly linked to tumor initiation and progression [6]. A structural analogue of resveratrol, pterostilbene (*trans*-3,5-dimethoxy-4-hydroxystilbene), has recently attracted interest due to its metabolic stability and significant pharmacological activities [7].

In a recent work, both the cytotoxicity and the ability to target duplex/G-quadruplex DNA of resveratrol **1**, pterostilbene **2** and their heterocyclic dimers (±)-*trans*-δ-viniferin (**3**) and (±)-pterostilbene-*trans*-dihydrodimer (**4**) have been investigated by our group [8]. All the tested compounds showed DNA-damaging activity consistent with their ability to interact with DNA structures and were cytotoxic at µM concentrations on a panel of cancer cell lines. Interestingly, (±)-*trans*-δ-viniferin (**3**) demonstrated higher affinity for the investigated DNA targets than its monomeric counterpart **1** [8].

Based on these results and considering that little is known about the bioactivities of resveratrol oligomers, we extended the cytotoxicity evaluation to other dimeric stilbenoids. Additionally, simplified analogues of the most promising compounds synthesized for a previous work [9] were tested to highlight the structural determinants responsible for the activity. Here, we report the results of this investigation.

## 2. Results and Discussion

### 2.1. Cytotoxic Activity Evaluation

**Figure 2:**
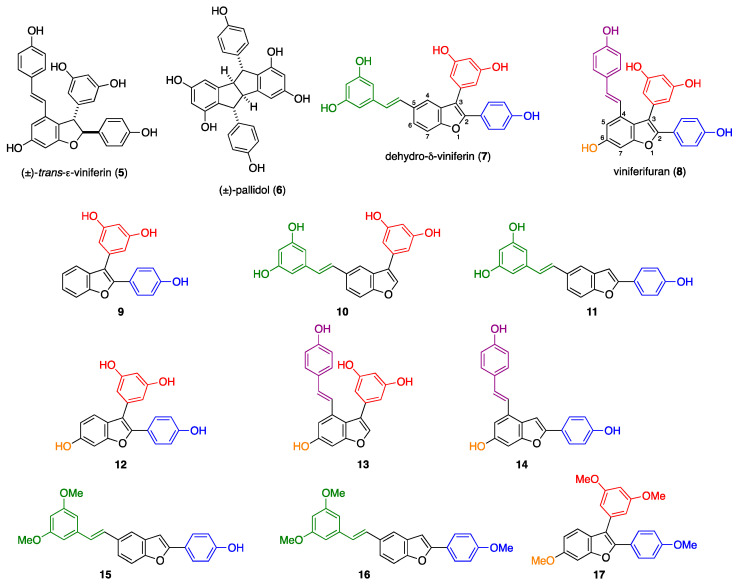
Structure of selected stilbenoid dimers **5**–**8** and simplified analogues of **7** (**9**–**11**, **15**, **16**) and **8** (**12**–**14**, **17**). The elicited groups are highlighted in blue (position 2), red (position 3), purple (position 4), green (position 5) and orange (position 6).

To obtain an integrated overview of representative stilbenoid dimers, natural compounds **3**–**6**, together with previously prepared viniferin dehydrodimers **7** and **8** (Figure 2), were assayed [10].

The antiproliferative activity of stilbenoid derivatives was evaluated upon 48 h exposure on melanoma A375, non-small cell lung cancer H460, PC3 prostate cancer cell lines and human normal skin WS1 fibroblasts, using the MTS cell proliferation assay (Table 1). Resveratrol **1** was used as a reference molecule and the IC_50_ values were determined as the concentrations of the compound causing 50% cell growth inhibition.

In order to provide a thorough overview of the antiproliferative activity, already published data about compounds **1**–**4** have been included in this discussion [8]. Although resveratrol **1** and pterostilbene **2** showed similar cytotoxicity, the former was more selective for tumour cells. The natural polyphenolic dimers (±)-δ-viniferin **3**, (±)-ε-viniferin **5** and (±)-pallidol **6** showed low antiproliferative activity against all the considered cell lines. Interestingly, the replacement of the 2,3-dihydrobenzofuran core of **3** and **5** with a benzofuran ring (compounds **7** and **8**) resulted in a 2–3-fold increased antiproliferative activity, although not accompanied by selectivity on tumor cells (**7** vs. **3** and **8** vs. **5**). On the other hand, the presence of the methoxy groups in **4** in place of the resorcinol moieties in **3** led to a significant increase in activity on tumor cell lines.

Comparing the cytotoxicity exerted on WS1 fibroblasts, the tetramethoxylated dihydrodimer **4** showed about 2-fold (PC3) and 3-fold (A375 and H460) higher antiproliferative activity on tumour cells. Conversely, polyphenolic dimers **3**, **5**, **6**, **7** and **8** showed a similar cytotoxic profile on all the tested cell lines.

To highlight the role of the various fragments of the most promising molecules **7** and **8**, a series of analogues with a simplified molecular backbone that have been recently synthesized for the evaluation of their antimicrobial activity [9] were tested (Figure 2). Compounds **9**, **10** and **11**, deriving from dehydro-δ-viniferin **7**, lacked the substituent in position 5 (styrene moiety, in green), 2 (phenol fragment, in blue) and 3 (resorcinol group, in red), respectively, of the benzofuran core (Figure 2). Removal of the aromatic ring at position 2 and 3 resulted in increased activity. In contrast, removal of the moiety in 5 led to decreased cytotoxicity. It is worth mentioning that all the three derivatives (**9**, **10**, **11**) showed an increased selectivity towards A375 and H460 tumour cells. The corresponding simplified analogue of compound **8**, i.e., **13** and **14**, showed an IC_50_ comparable to the parent compound, whereas removal of the styrene portion led again to a less active compound (**12**), similar to the previous series (Figure 2, Table 1).

Subsequently, the effect of the methylation of phenolic groups was evaluated, considering the significant increase in activity obtained by partial methylation of (±)-δ-viniferin **3** to give (±)-pterostilbene dimer **4**. Interestingly, compound **15,** bearing two methoxy groups on the styrene portion, significantly improved the activity against A375 and H460 cells compared to trihydroxylated compound **11**, whereas permethylated **16** and **17** did not show cytotoxic activity (Figure 2, Table 1).

### 2.2. DNA Damage

**Figure 3:**
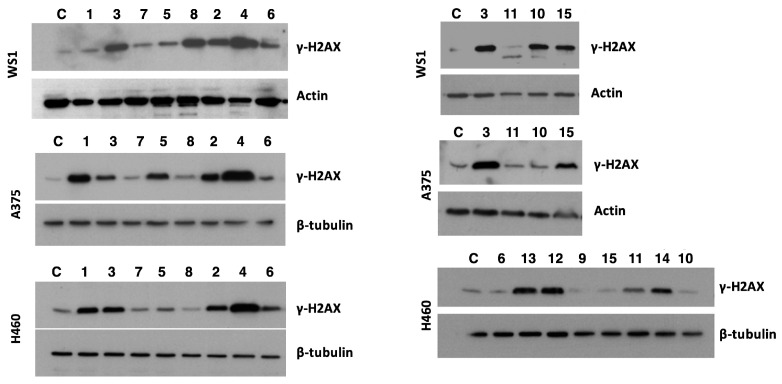
Twenty-four hours after seeding, WS1, A375 and H460 cells were exposed to compounds for 48 h at concentrations corresponding to IC_50_ at 48 h. Cells were then harvested and processed for Western blot assay. Tubulin and actin represent the control for loading; for compounds **1**–**4,** see ref. [8].

Among the DNA lesions, double-strand breaks (DSBs) represent the most dangerous breaks for the cells. Unrepaired DSBs result in genomic instability and chromosome aberrations, ultimately leading to cell death. DSBs can be induced by chemical, physical and biological factors and, once produced, activate the DNA damage response machinery, which recognizes and repairs the damage. At the molecular level, a pivotal role is played by the phosphorylation of histone H2AX, leading to γ-H2AX. Phosphorylated γ-H2AX favours the accumulation of proteins involved in DNA repair at the sites of damaged chromatin. This leads to the activation of checkpoint proteins and, subsequently, to the arrest of cell cycle progression. The evaluation of the levels of γ-H2AX is useful for detecting the DNA damage caused by toxic compounds, including anticancer drugs operating through a DNA damage-dependent mechanism of action [11]. To evaluate the ability of the selected dimers and the most potent simplified analogues to produce DNA damage, the expression of γ-H2AX after compound exposure was evaluated in WS1, H460 and A375 cell lines in comparison with monomers **1** and **2** (Figure 3). Cells were exposed for 48 h to the studied compounds at a concentration corresponding to their respective IC_50_ and the level of γ-H2AX was evaluated by Western blot assay (Figure 3). No DNA damage was evidenced in normal WS1 fibroblasts exposed to 200 µM of resveratrol **1**, whereas in H460 and A375 cells, the compound showed significant damaging activity. This behaviour confirms the results obtained in a previous work, demonstrating that even in the high micromolar range, resveratrol **1** is selective towards cancer cells, with no effect on normal fibroblasts [12]. (±)-*trans*-δ-Viniferin **3** and (±)-pterostilbene **2** produced DNA damage in all the cell lines, including WS1 cells, whereas compounds **5**, **6** and **7** had negligible effects on all the considered cell lines. (±)-Pterostilbene-*trans*-dihydrodimer **4** emerged as the most active DNA damaging compound of the series, although demonstrating low selectivity (Figure 3).

It is worth noting that the replacement of the 2,3-dihydrobenzofuran core of **3** and **5** with a benzofuran ring (compounds **7** and **8**) led to an increase in cytotoxic activity, which did not correspond to an increase in DNA damage. However, this behaviour could be explained by a different molecular target of the oxidized compounds compared to the parent ones.

Besides its rather selective cytotoxic activity, compound **10** induced significant DNA damage in human WS1 cells, but not in H460 and A375 cancer cells. Conversely, compound **11** produced slight DNA damage only in H460 cells. Methylation of the resorcinol moiety of compound **11** to give **15** increased the DNA damage in WS1 and A375 cells. 

To have a broader overview, compounds **9** and **12**–**14**, endowed with lower cytotoxic activity, were tested to evaluate DNA damage in H460 cells. 6-OH derivatives **12**–**14** had significant damaging activity, whereas compound **9**, lacking the hydroxyl on the benzofuran ring, did not show any relevant effect. Such a difference in terms of damaging activity could suggest that, in the case of the simplified analogues, the OH group on the benzofuran core is involved in DNA damage.

## 3. Materials and Methods

### 3.1. Synthesis

Natural compounds **3**–**6** together with viniferin dehydrodimers **7** and **8** were synthesized following a previously reported procedure by our research group [10]. In a similar way, viniferin analogues **9**–**17** were prepared by the synthetic approach described in ref. [9]. A schematic representation of the syntheses is reported in the Supplementary Materials (Schemes S1–S7).

### 3.2. Cell Lines

The human skin normal WS1 fibroblasts (ATCC CRL-1502) were cultured in Eagle’s Minimum Essential Medium plus 10% FBS. The human malignant melanoma A375 cells (ATCC CRL-1619) were cultured in Dulbecco’s Modified Eagle’s Medium plus 10% FBS. The non-small cell lung cancer NCI-H460 cells (ATCC HTB-177) and the prostate cancer PC-3 cells (ATCC CRL-1435) were cultured in RPMI 1460 plus 10% FBS. All the cell lines were grown at 37 °C and 5% CO_2_.

### 3.3. Cytotoxicity Assay

Cytotoxic activity was assessed by growth inhibition assay (CellTiter 96^®^ AQueous One Solution Cell Proliferation Assay MTS, Promega, Madison, WI, USA). Twenty-four hours after seeding in 96-well plates, cells were exposed to the selected molecules (1–100/200 μM) for 48 h. At the end of the treatment, MTS solution (20 μL) was added to each well. The FLUOstar OPTIMA plate reader (BMG Labtech GmbH, Offenburg, Germany) was used to measure the absorbance at 492 nm after 4 h incubation in 5% CO_2_ at 37 °C. Dose–response curves were used to define IC_50_ values as the compound concentration causing 50% cell growth inhibition. Experiments were performed in triplicate.

### 3.4. Western Blot Assay

Cells were seeded in 6-well plates and 24 h later, they were exposed for 48 h to the compounds at concentrations corresponding to their IC_50_ values. Cells were harvested and lysated. Samples were fractionated on SDS-PAGE and transferred to a nitrocellulose film. A solution of non-fat dry milk (5% *w*/*v*) was used to block the membranes before incubating with the primary antibody. Following the incubation with the HRP-conjugated secondary antibody, films were developed by chemiluminescence (ECL, Amersham, UK). The levels of actin and β-tubulin were used as the control for loading.

## 4. Conclusions

Monomeric stilbenoids are under intensive investigation as anticancer compounds; however, little is known regarding the cytotoxicity of their oligomers. Four natural resveratrol and pterostilbene dimers, i.e., (±)-*trans*-δ-viniferin **3**, (±)-pterostilbene dihydrodimer **4**, (±)-ε-viniferin **5** and (±)-pallidol **6,** were investigated to shed light on their cytotoxic potential. Reduced cytotoxicity, together with low selectivity, were observed for resveratrol dimers **3**, **5** and **6**, compared to resveratrol **1**. Conversely, cytotoxic potency was recovered by the pterostilbene dimer **4**. Additionally, compound **4** showed higher selectivity towards tumour cell lines and significant DNA-damaging activity. All the reported data confirmed the importance of the methylation of phenolic groups in increasing the activity as already observed for the monomers (see pterostilbene **2** vs. resveratrol **1**). 

Worthy of note was the improvement of the antiproliferative activity following the replacement of the dihydrobenzofuran core of the viniferins with a benzofuran ring (compounds **7** and **8** vs. **3** and **5**). This evidence prompted us to investigate the role played by the substituents of the benzofuran backbone. Thus, a small collection of simplified analogues of the most promising compounds was synthesized to highlight the structural determinants responsible for the activity.

It was observed that the methylation of the phenol moiety of compound **11** improved the cytotoxicity in compound **15**. At the same time, the methylation of the three OH groups of **11** and **12** (compounds **16** and **17**) totally abolishes the compound potency, leading to the conclusion that a phenol group in position 2 or 3 of the benzofuran ring is pivotal for the activity. The introduction of a OH residue on C-6 had no effect on the compound potency (**9** vs. **12**), although higher DNA damage capability was observed. This holds true also for compounds **13** and **14**. Overall, the gathered data highlight compound **15** as the most promising candidate to undergo a structural optimization campaign.

Based on the obtained findings, interaction with DNA could be considered one of the anticancer mechanisms of action of dimeric stilbenoids and their simplified analogues. However, additional mechanisms involving other targets cannot be excluded, in accordance with previous reports in the literature [12,13].

The design and synthesis of novel benzofuran-containing compounds based on natural stilbenoids are being actively undertaken in our laboratories to shed light on the intriguing anticancer profile of this multifaceted class of compounds.

## Figures and Tables

**Table 1:** Cytotoxic activity of selected stilbenoids ^a^.

Cpd	WS1	A375	H460	PC3
**IC_50_ (µM) ^b^**				
**1 ^c^**	>200	44.5 ± 3.5	25 ± 0.77	>100
**2 ^c^**	57 ± 10	33 ± 0.7	25 ± 0.4	97 ± 4.9
**3 ^c^**	69 ± 5.6	95 ± 7	81 ± 4	120 ± 7.8
**4 ^c^**	82.7 ± 1.1	25.5 ± 2.1	24.7 ± 0.3	46.7 ± 3.3
**5**	82 ± 2.8	95 ± 2.8	61.3 ± 17	95 ± 0.2
**6**	>100	93 ± 2.8	73 ± 0.3	>200
**7**	37 ± 1.4 ^d^	42.3 ± 8.0	27 ± 0.3	46 ± 0.5
**8**	33 ± 1.4 ^d^	46.5 ± 3.5	26 ± 1.0	43.3 ± 0.4
**9**	98.7 ± 1.8 ^d^	58.2 ± 0.8	50 ± 3.5	101 ± 1.4
**10**	96.8 ± 4.5 ^d^	24 ± 1.8	28 ± 2.8	100 ± 0.7
**11**	97.8 ± 3.0 ^d^	28 ± 9	57 ± 6.4	86 ± 2.8
**12**	98.5 ± 2.0 ^d^	67 ± 1.9	77 ± 0.42	99 ± 1.4
**13**	85 ± 4.6 ^d^	36 ± 1.1	69 ± 0.3	98 ± 2
**14**	45 ± 1.2 ^d^	46 ± 3.5	26.8 ± 0.3	42.7 ± 0.5
**15**	95 ± 2.3 ^d^	18 ± 2.8	25 ± 2.1	85 ± 21
**16**	>100 ^d^	>100	>100	>100
**17**	>100 ^d^	>100	>100	>100

^a^ Twenty-four hours after seeding, cells were exposed for 48 h to the compounds and cytotoxicity was measured using MTS assay. Data represent mean values ± SD of three independent experiments; ^b^ IC_50_ is defined as the concentration of the compound causing 50% cell growth inhibition; for the IC_50_ of compounds **1**–**4**, see ref. [8]; ^c^ model compounds included for comparison purposes, ref. [8]; ^d^ preliminary evaluation on WS1 in ref. [9].

## Data Availability

Data is contained within the article or Supplementary Material.

## Supplementary Materials

The following supporting information can be downloaded at: https://www.mdpi.com/article/10.3390/ph17081012/s1, Table S1: Chemicalize was used for the calculation of the physico-chemical properties for all the tested compounds, March 2024, https://chemicalize.com/, developed by ChemAxon; Schemes S1–S3: Synthesis of compounds **3**–**9**; Schemes S4–S6: Synthesis of compounds **10**–**13** and **15**–**17**; Scheme S7: Synthesis of compound **14**.
