# Supplementary material for: Cytotoxicity of Benzofuran-Containing Simplified Viniferin Analogues
Source: Pharmaceuticals (Basel). 2024 Aug 1;17(8):1012. doi: 10.3390/ph17081012 (PMC11357204; doi:10.3390/ph17081012)
Supplement: Supplementary file 1 [file pharmaceuticals-17-01012-s001.zip › pharmaceuticals-3083430-Supplementary Material.pdf]

Cytotoxicity of benzofuran-containing simplified viniferin analogues

Salvatore Princiotto [1,*], Cecilia Pinna [1], Luce Micaela Mattio [1], Francesca Annunziata [1], Giovanni Luca Beretta [2], Andrea Pinto [1] and Sabrina Dallavalle [1]

[1]Department of Food, Environmental and Nutritional Sciences (DeFENS), University of Milan, Via Celoria 2, 20133, Milan, Italy; e-mail: cecilia.pinna@unimi.it (C.P.); luce.mattio@unimi.it (L.M.M.); francesca.annunziata@unimi.it (F.A.); andrea.pinto@unimi.it (A.P.); sabrina.dallavalle@unimi.it (S.D.)
[2]Molecular Pharmacology Unit, Department of Experimental Oncology, Fondazione IRCCS Istituto Nazionale Tumori, via Amadeo 42, 20133 Milan, Italy; email: giovanni.beretta@istitutotumori.mi.it (G.L.B.)

*Correspondence: salvatore.princiotto@unimi.it

Table S1. Calculated physico-chemical properties Page S2

Scheme S1-S3. Synthesis of compounds **3-9** Page S3

Scheme S4-S6. Synthesis of compounds **10-13** and **15-17** Page S4

Scheme S7. Synthesis of compound **14** Page S5

Table S1. Chemicalize was used for the calculation of the physico-chemical properties for all the tested compounds, March 2024, https://chemicalize.com/, developed by ChemAxon.

compound	Molar mass	cLogP	nHA	nHD	HLB	TPSA	VDW volume
**1**	228.08	3.40	3	3	8.66	60.69	204.56
**2**	256.11	3.69	3	1	7.91	38.69	239.77
**3**	454.14	5.96	6	5	9.36	110.38	394.53
**4**	510.20	6.54	6	1	7.29	66.38	465.54
**5**	454.48	5.96	6	5	9.36	110.38	394.32
**6**	454.48	5.31	6	6	8.93	121.38	389.5
**7**	452.46	6.17	5	5	8.81	130.41	387.01
**8**	452.46	6.17	5	5	8.81	114.29	386.91
**9**	318.33	4.44	3	3	7.45	73.83	272.54
**10**	360.37	4.91	4	4	8.89	94.06	307.92
**11**	344.37	5.13	3	3	7.21	73.83	299.59
**12**	334.33	4.13	4	4	9.14	94.06	281.19
**13**	360.36	4.91	4	4	8.89	94.06	307.96
**14**	344.37	5.13	3	3	7.21	73.83	299.36
**15**	372.42	5.42	3	1	6.30	51.83	334.71
**16**	386.45	5.57	3	0	5.83	40.83	352.03
**17**	390.44	4.72	4	0	7.17	50.06	351.54

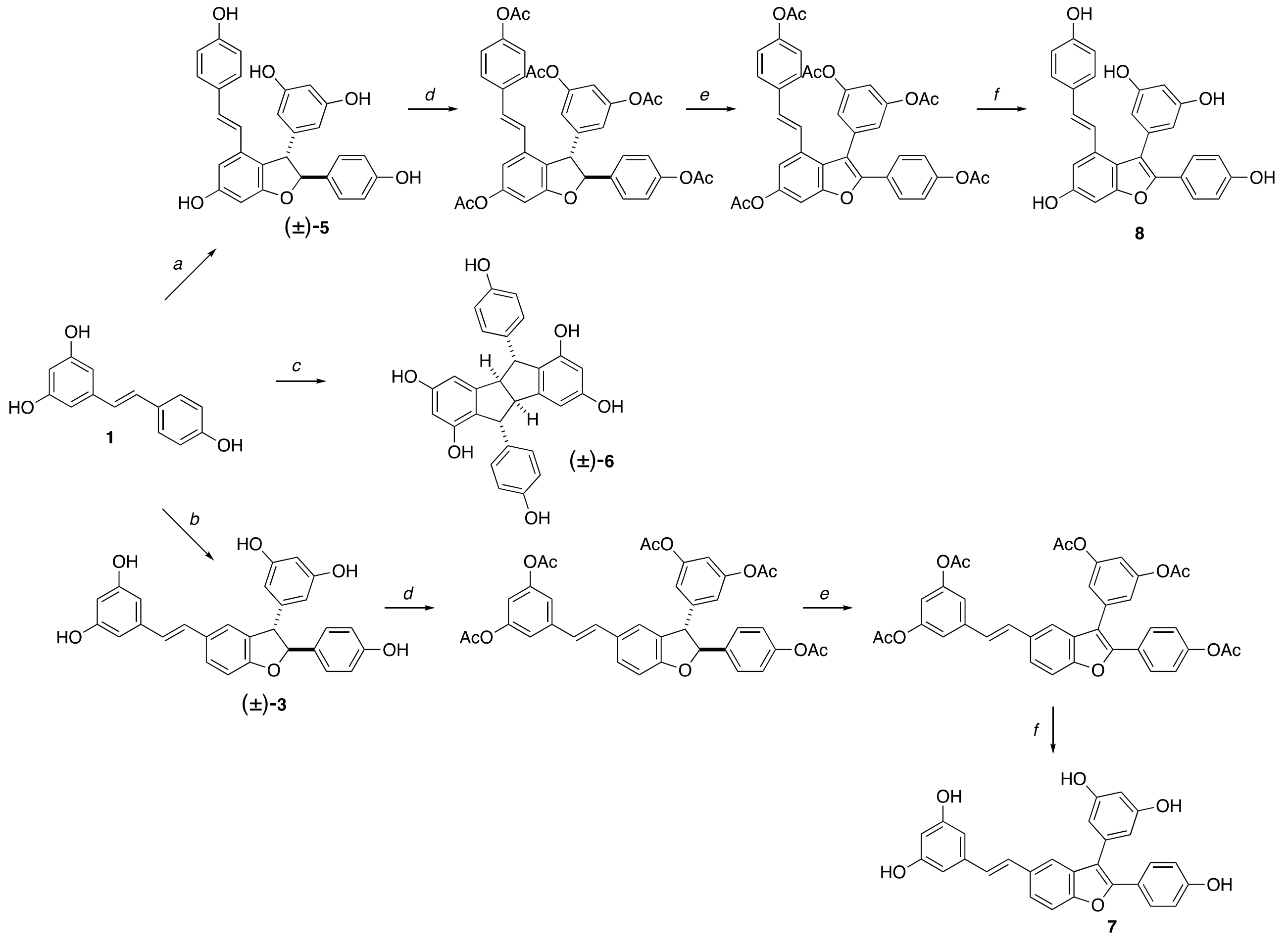

Scheme S1. Synthetic pathway for the obtainment of (±)-**3**, (±)-**5**, **7** and **8**. *a*. $FeCl_3$ $6H_2O$, MeOH, rt, **15%**; *b.* i. HRP, 40 °C, acetone/citrate buffer pH 5 1:1; ii. H_2O_2, 40 °C, **49%** (over two steps); *c.* i. HRP, 40 °C, acetone/phosphate buffer pH 8 1:1; ii. H_2O_2, 40 °C, **21%** (over two steps); *d.* Ac_2O, TEA, DCM/DMSO, rt, **90-92%**; *e.* DDQ, DCM, reflux, **60-84%**; *f.* KOH, MeOH, 0 °C, **55-70%**

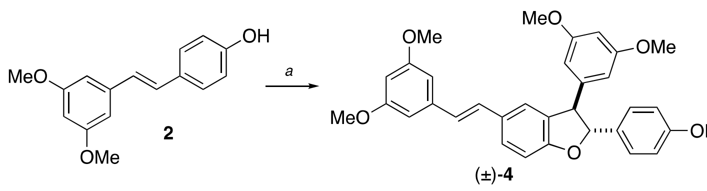

Scheme S2. Synthetic pathway for the obtainment of (±)-**4**. *a*. i. HRP, 30 min, 40 °C, acetone/citrate buffer pH 5 1:1; ii. H_2O_2, 15 min, 40 °C, **60%** (over two steps)

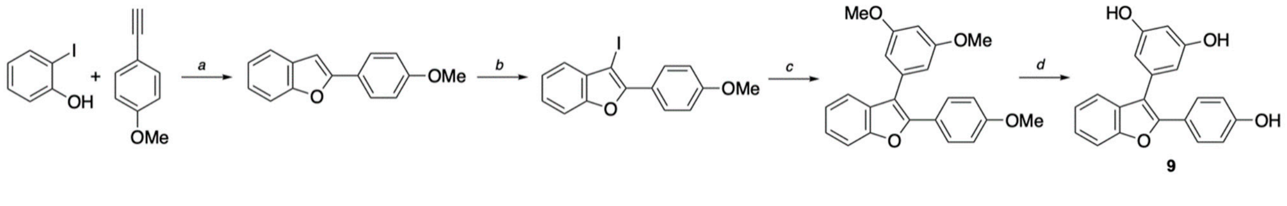

Scheme S3. Synthetic pathway for the obtainment of **9**. *a*. CuI, $PdCl_2(PPh_3)_2$, TEA, THF/MeCN, 100 °C, **50%**; *b.* NIS, PTSA, MeCN, rt, **74%**; *c.* 3,5-dimethoxyphenyl boronic acid, K_2CO_3, $PdCl_2(dppf)$, THF/H_2O, 70 °C, **83%**; *d.* BBr_3, DCM, 0 °C to rt, **61%**

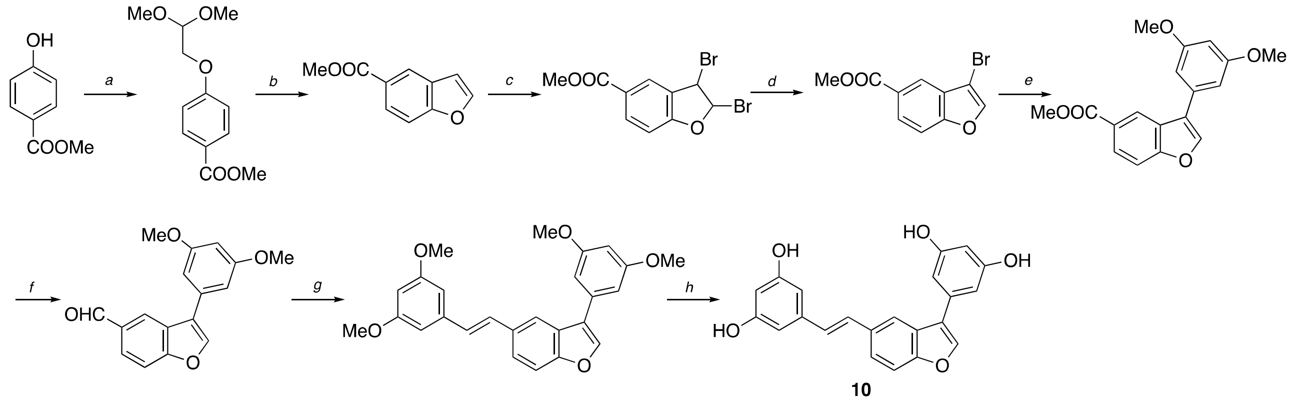

Scheme S4. Synthetic pathway for the obtainment of **10**. *a*. 2-Br-1,1-DME, Cs_2CO_3, MeCN, reflux, **61%**; *b*. amberlyst-15, toluene, reflux, **51%**; *c*. Br_2, DCM, 0 °C to rt, **82%**; *d*. KOH, MeOH/THF, 0 °C, **82%**; *e*. 3,5-dimethoxyphenylboronic acid, $Pd(PPh_3)_4$, Na_2CO_3, DME/H_2O, 80 °C, **74%**; *f*. i. $LiAlH_4$, THF, 0 °C, **97%**; ii. DMP, DCM, 0 °C to rt, 78%; *g*. diethyl (3,5-dimethoxyphenyl)phosphonate, NaH, THF, MW, 120 °C, **80%**; *h*. BBr_3, DCM, 0 °C to rt, **14%**

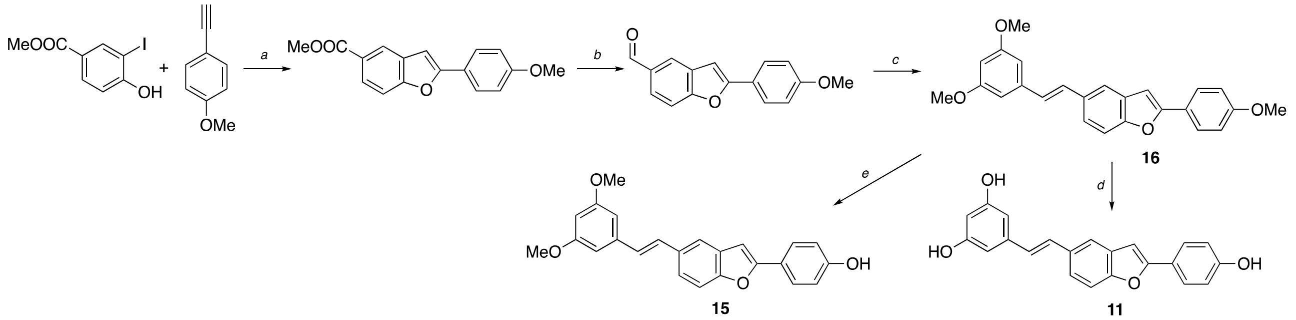

Scheme S5. Synthetic pathway for the obtainment of **11**, **15** and **16**. *a*. CuI, $PdCl_2(PPh_3)_2$, TEA, THF/MeCN, 100 °C, **62%**; *b*. $LiAlH_4$, THF, 0 °C, **85%**; *c*. 3,5-dimethoxyphenyl phosphonate, NaH, THF, MW, 120 °C, **76%**; d. BBr_3, DCM, 0 °C to rt, **60%**; *e*. BCl_3, TBAI, DCM, 0 °C to rt, **35%**

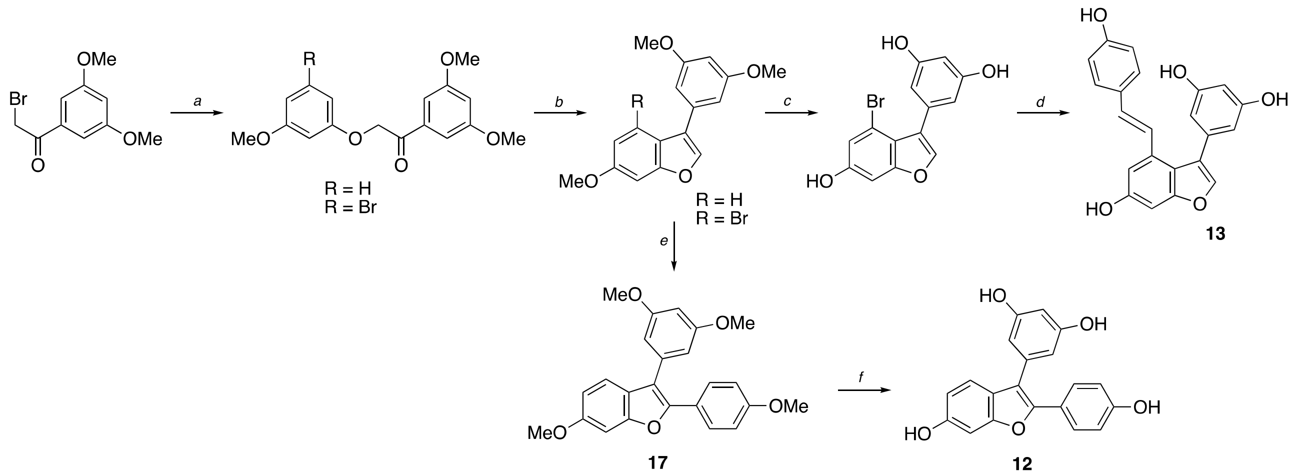

Scheme S6. Synthetic pathway for the obtainment of **12**, **13** and **17**. *a*. 3-methoxyohenol (R = H) or 3-Br-5-methoxyphenol (R = Br), K_2CO_3, acetone, reflux, **90%** (R = H) or **89%** (R = Br); *b*. $Bi(OTf)_3$, DCM, reflux, **43%** (R = H) or **83%** (R = Br); *c*. BBr_3, DCM, -78 °C to rt, **91%**; *d*. *p*-Hydroxystyrene, $Pd(OAc)_2$, TEA, dppp, DMF, 120 °C, **80%**; *e*. 4-Br anisole, $Pd(OAc)_2$, PCy_3HBF_4, K_2CO_3, pivalic acid, DMA, 100 °C, **80%**; *f*. BBr_3, DCM, -78 °C to rt, **52%**

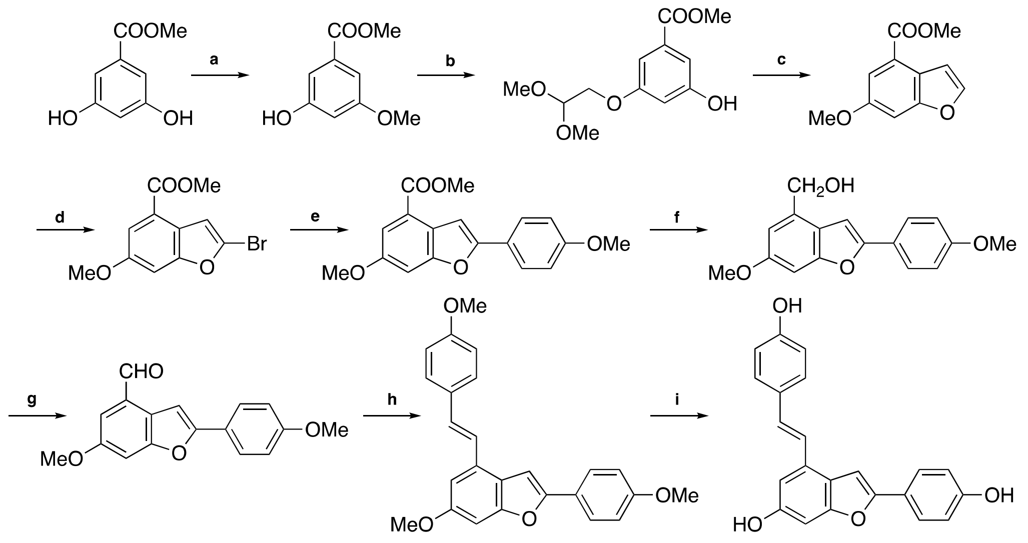

Scheme S7. Synthetic pathway for the obtainment of **14**. *a*. MeI, K_2CO_3, DMF, rt, **35%**; *b*. 2-Br-1,1-DME, Cs_2CO_3, MeCN, reflux, **67%**; *c*. amberlyst-15, PhCl, 120 °C, **63%**; *d*. NBS, 1,2-DCE/DMF, 70 °C, 80%; *e*. 4-OMe-PBA, K_2CO_3, $Pd(PPh_3)_4$, 70 °C, **91%**; *f*. $LiAlH_4$, THF, 0 °C, **89%**; *g*. DMP, DCM, **97%**; *h*. diethyl (4-OMe-Bn) phosphonate, NaH, MW, 120 °C, **54%**; *i*. BBr_3, DCM, **24%**
